# Case Report: A comprehensive digital workflow to enhance predictability and precision with fixed dental prostheses in the posterior region

**DOI:** 10.3389/fdmed.2025.1625405

**Published:** 2025-06-24

**Authors:** Isabela Toser, Andrei-Bogdan Faur, Anca-Elena Anghel-Lorinți, Anca Jivănescu

**Affiliations:** ^1^ Department of Prosthodontics, University of Medicine and Pharmacy “Victor Babes”, Timisoara, Romania; ^2^ Department of Prosthodontics, TADERP Research Center, University of Medicine and Pharmacy “Victor Babes”, Timisoara, Romania

**Keywords:** digital prosthodontics, occlusal analysis, intraoral scanning, fixed partial dentures, digital workflow, facial scanning, prosthetic rehabilitation

## Abstract

This clinical report presents a full digital protocol for prosthetic rehabilitation following the bilateral loss of maxillary first molars. The aim of the study was to explore the integration of advanced digital technologies, including intraoral scanning, facial scanning, cone-beam computed tomography (CBCT), and digital occlusal analysis, into a comprehensive treatment protocol for the fabrication of fixed partial dental prostheses. A key focus was on evaluating the precision of occlusal equilibration using digital occlusal analysers in conjunction with conventional articulating paper. While the design was sent to a dental laboratory for fabrication, the workflow demonstrated efficiency, minimal invasiveness, and a high degree of predictability in achieving both functional and aesthetic outcomes, mostly manageable in a chair-side manner. The results showed that most occlusal contacts translated successfully from virtual planning to the intraoral environment; however, some discrepancies were noted, which could be attributed to the absence of certain motion data in the digital workflow. The integration of digital occlusal analysis was essential in identifying and adjusting premature contacts, contributing to improved patient comfort and occlusal stability. The study highlights the potential of a fully digital workflow in modern prosthodontics, emphasizing its role in achieving more predictable outcomes, enhancing clinical efficiency, and reducing the need for post-cementation adjustments. Future research should explore.

## 1 Introduction

Current advancements in digital dentistry indicate that clinical and laboratory procedures are progressively being supplanted by digital workflows. The traditional prosthesis manufacturing method involves impression taking, waxing, try in, casting, and polishing. This procedure necessitates substantial effort from doctors and laboratory professionals, incurs significant expenses, involves prolonged fabrication durations, and carries the risk of impression distortion or cast deterioration. Since its beginning in the late 1980s, computer-aided design and computer-aided manufacture (CAD-CAM) technology has facilitated more efficient workflows for clinicians and laboratory technicians, enhanced quality control, decreased costs and production time, and enabled the utilization of innovative materials such as zirconia (1–4).

Digital technologies, including as intraoral scanners (IOSs) or optical jaw tracking devices, can be utilized in prosthodontic treatment (5, 6). The precision of IOSs can be optimized by comprehending the operator as well as patient-related variables that may diminish scanning accuracy (7). Articulated intraoral scans serve as the foundation for jaw tracking systems that facilitate the documentation of the envelope of function (8). The recorded mastication area in a patient with satisfactory function offers crucial functional data that can be included in the virtual design for restorations. Traditional techniques employ articulating paper to assess and modify the occlusal contacts of prosthesis during mastication. Jaw tracking devices can fulfil the similar role in prosthesis design, hence minimizing delivery time (9).

The evolution of digital technologies has profoundly transformed modern dental practice, particularly in prosthetic rehabilitation. Innovations such as intraoral scanning, face scanning, CAD/CAM systems, 3D printing, and digital occlusal analysis have contributed to workflows that are faster, more accurate, and more predictable compared to conventional approaches (9, 10). Alongside these technological advancements, research has increasingly focused on the selection and performance of dental materials, clinician preferences, and the optimization of restorative designs to enhance clinical outcomes (11, 12).

Recent studies have provided valuable insights into current patterns of material use among dental practitioners, emphasizing the growing preference for adhesive systems and bio ceramic-based materials that align with minimally invasive, biologically integrated approaches (11, 13). Furthermore, the structural integrity of restorative options, such as zirconia-based fixed partial dentures, has been shown to be significantly influenced by prosthetic design factors like pontic length and occlusal forces (14). In complex rehabilitations, particularly for patients with periodontal compromise, long-term success depends not only on material selection but also on precise occlusal management and restorative planning (15).

Within this context, integrating digital tools for diagnosis, treatment planning, and functional assessment—including emerging technologies like facial scanning—offers new opportunities for achieving both aesthetic and functional excellence.

This clinical report demonstrates the application of a fully digital workflow for the diagnosis, treatment planning, and fabrication of two fixed dental prostheses, following the bilateral loss of maxillary first molars. The goal of this case is to highlight the clinical benefits of integrating advanced digital tools—including intraoral scanning, facial scanning, CBCT, and digital occlusal analysis—into the treatment process. Additionally, this study explores the role of digital occlusal analysers as a complementary tool to conventional articulating paper, aiming to achieve precise occlusal equilibration while minimizing the need for post-cementation adjustments.

## 2 Materials and methods

### 2.1 Ethics, case introduction, and clinical objectives

This clinical case was approved by the Research Ethics Committee of the “Victor Babeș” University of Medicine and Pharmacy, Timișoara (Approval No. 72/20.12.2024) and conducted in accordance with the principles of the Declaration of Helsinki. The patient provided written informed consent for participation in the study, including agreement for the use of clinical data and photo-video documentation.

A 30-year-old female patient presented to the Department of Prosthodontics, Faculty of Dental Medicine, “Victor Babeș” University of Medicine and Pharmacy, seeking prosthetic rehabilitation for missing maxillary first molars (teeth 1.6 and 2.6). Despite being informed about implant-supported prosthetic options, the patient declined implant therapy and opted instead for fixed dental prostheses on natural abutment.

In addition to restoring functional occlusion, the patient emphasized the importance of achieving an aesthetically pleasing outcome. Another goal of this clinical case was to evaluate importance of digital tools in the evaluation of the accuracy of occlusal contacts before and after insertion of the fixed dental prostheses. Specifically, the objective was to determine whether the occlusal contact points visualized during the CAD design phase would be accurately replicated intraorally after final cementation—without requiring any occlusal adjustments. The underlying hypothesis was that, with a fully digital workflow and minimal protocol error, the final intraoral contacts would mirror those of the digital design, both on the restorations and adjacent natural dentition.

### 2.2 Initial clinical examination and occlusal evaluation

Clinical examination revealed that teeth 1.7, 1.5, and 1.4 had previously undergone endodontic treatment and had already been prepared as abutments during an earlier, unfinished treatment. Teeth 2.5 and 2.7 were vital and restored with composite fillings.

A thorough intraoral evaluation was performed, with no clinical signs of periodontal disease. Occlusal analysis was conducted using the OccluSense system (Bausch GmbH, Cologne, Germany), which identified premature contacts on the mandibular right third molar. These interferences were corrected through selective enamel grinding to establish a functional occlusal plane prior to initiating the digital diagnostic workflow.

### 2.3 Digital diagnostic protocol and smile design

A comprehensive digital workflow was implemented to evaluate the initial condition and plan the prosthetic treatment. The diagnostic protocol included intraoral and extraoral photography, intraoral scanning, facial scanning, and panoramic radiography. Additionally, the patient provided a recent cone-beam computed tomography (CBCT) scan (Planmeca ProMax® 3D Mid, Planmeca Oy, Helsinki, Finland), which was integrated into the digital evaluation.

**Figure 1:**
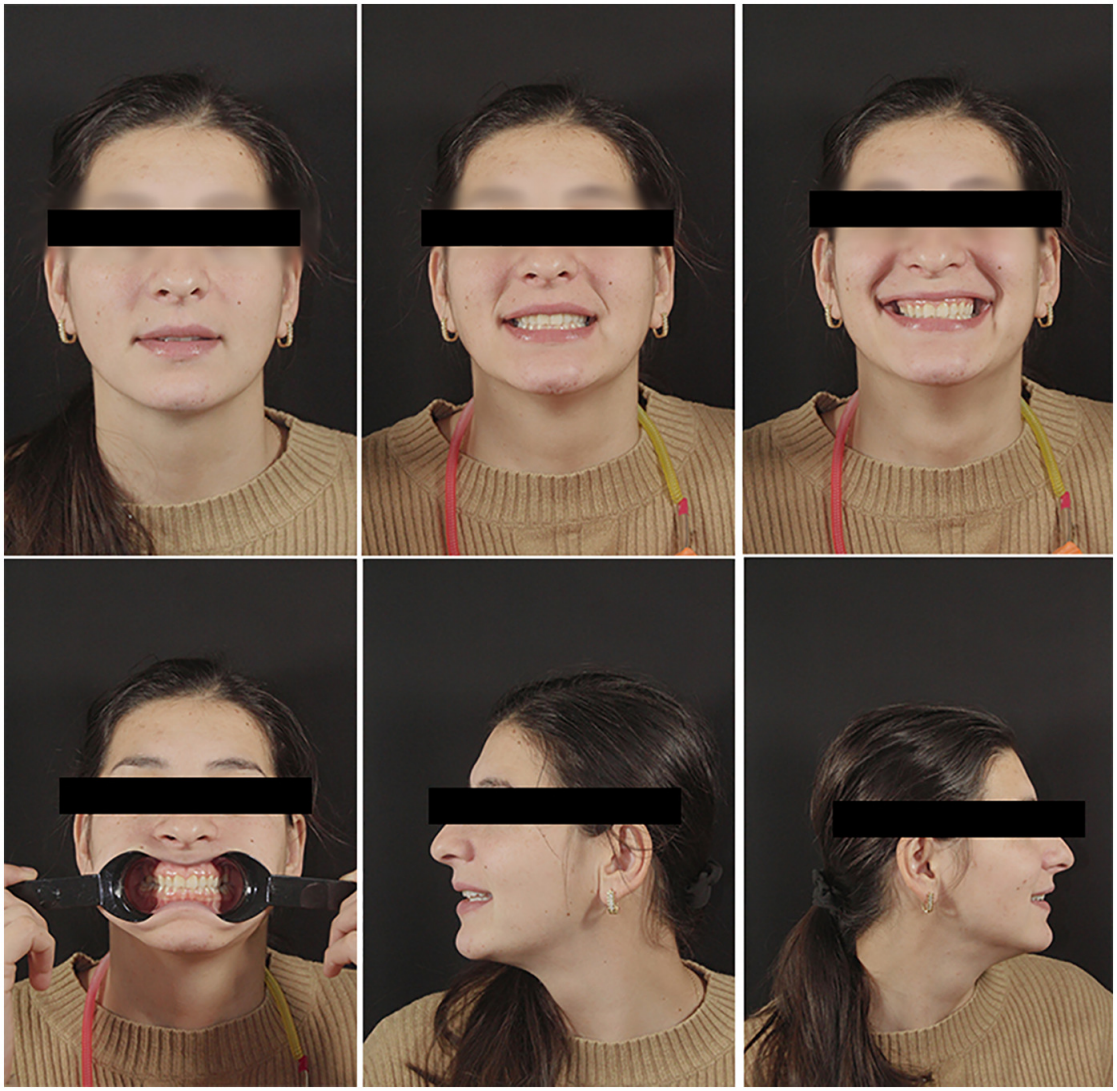
Extraoral photographs of the patient.

**Figure 2:**
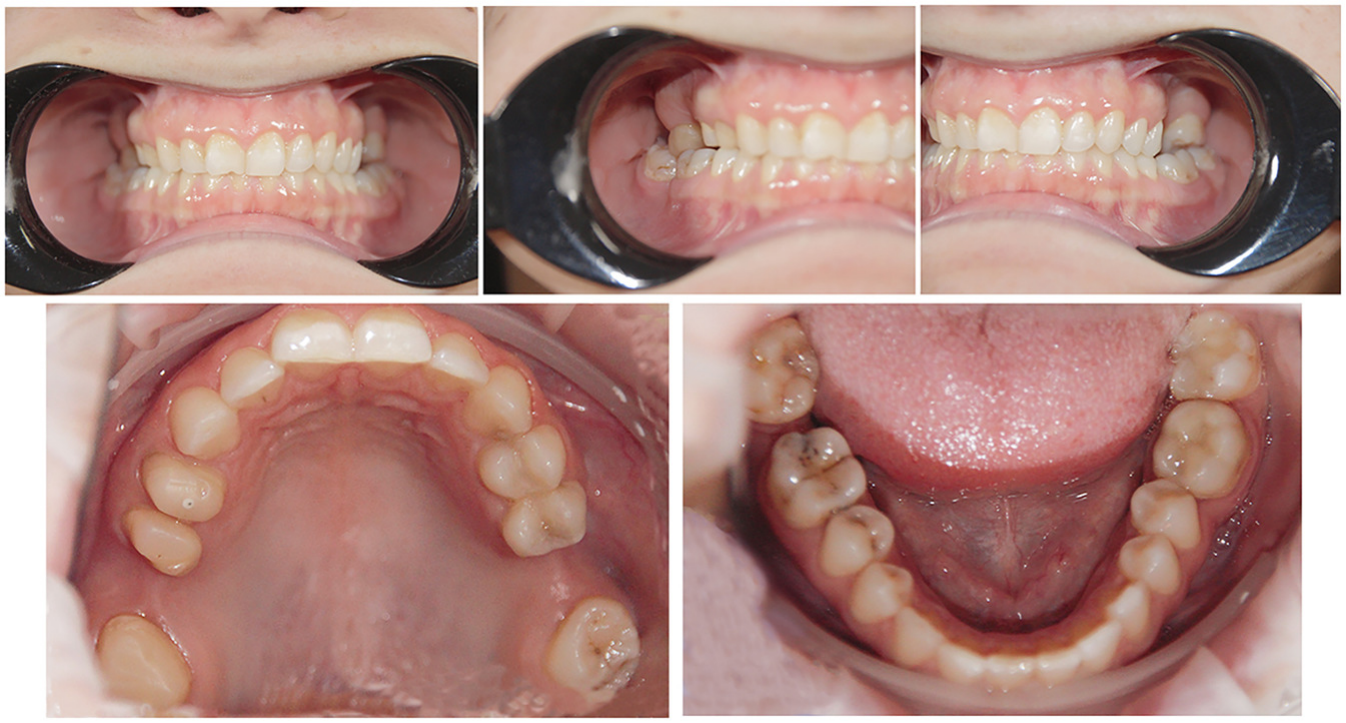
Intraoral photographs of the patient.

**Figure 3:**
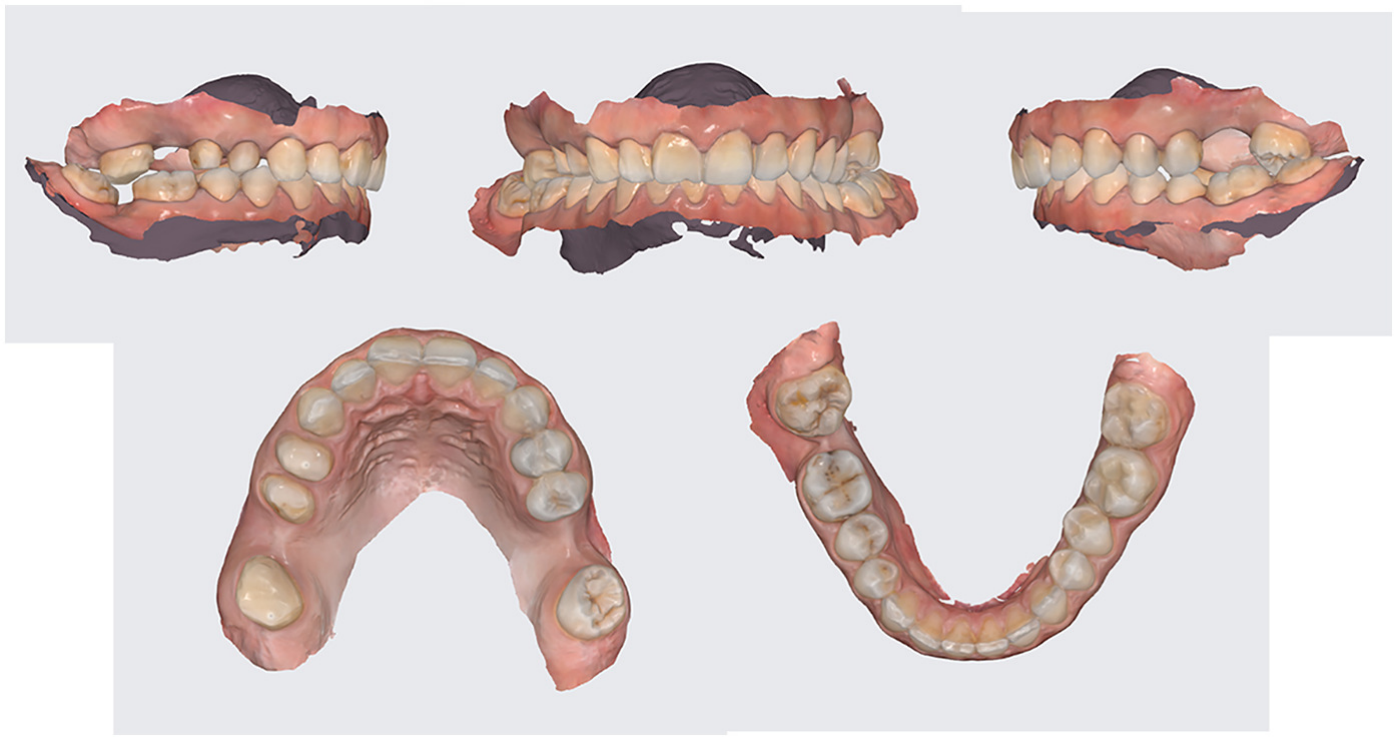
Intraoral scans of the initial situation.

**Figure 4:**
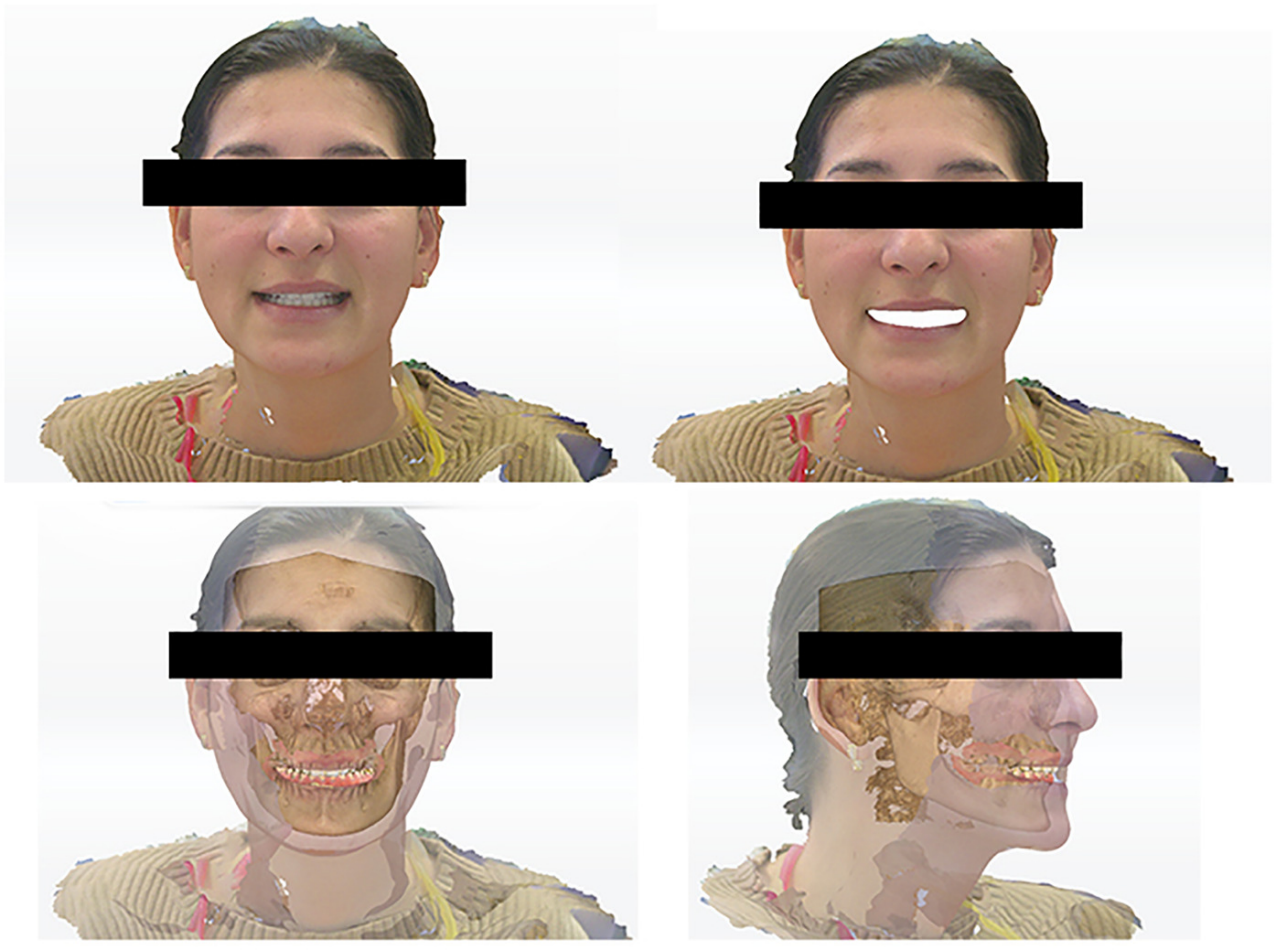
Facial scan of the patient aligned with the intraoral scans and the CBCT investigation of the patient.

Extraoral photographs were captured using a DSLR camera (Nikon D7500, with AF-S Micro Nikkor 85 mm lens and Fixlite Twin Softbox flash system) (Figure 1). Intraoral photographs were acquired using the same setup (Figure 2). Digital impressions and bite registration were obtained using the Medit i700 intraoral scanner (Medit Corp., Seoul, South Korea) (Figure 3). A facial scan was performed using the MetiSmile facial scanner (Shining 3D, Hangzhou, China) (Figure 4). During the facial scanning protocol, the intraoral scans were imported and aligned with the facial mesh for accurate integration. The CBCT scan was also registered and superimposed with both the intraoral scans and the facial scan using the proprietary alignment functions of the Shining 3D software, resulting in a fully digitized virtual patient (Figure 4).

**Figure 5:**
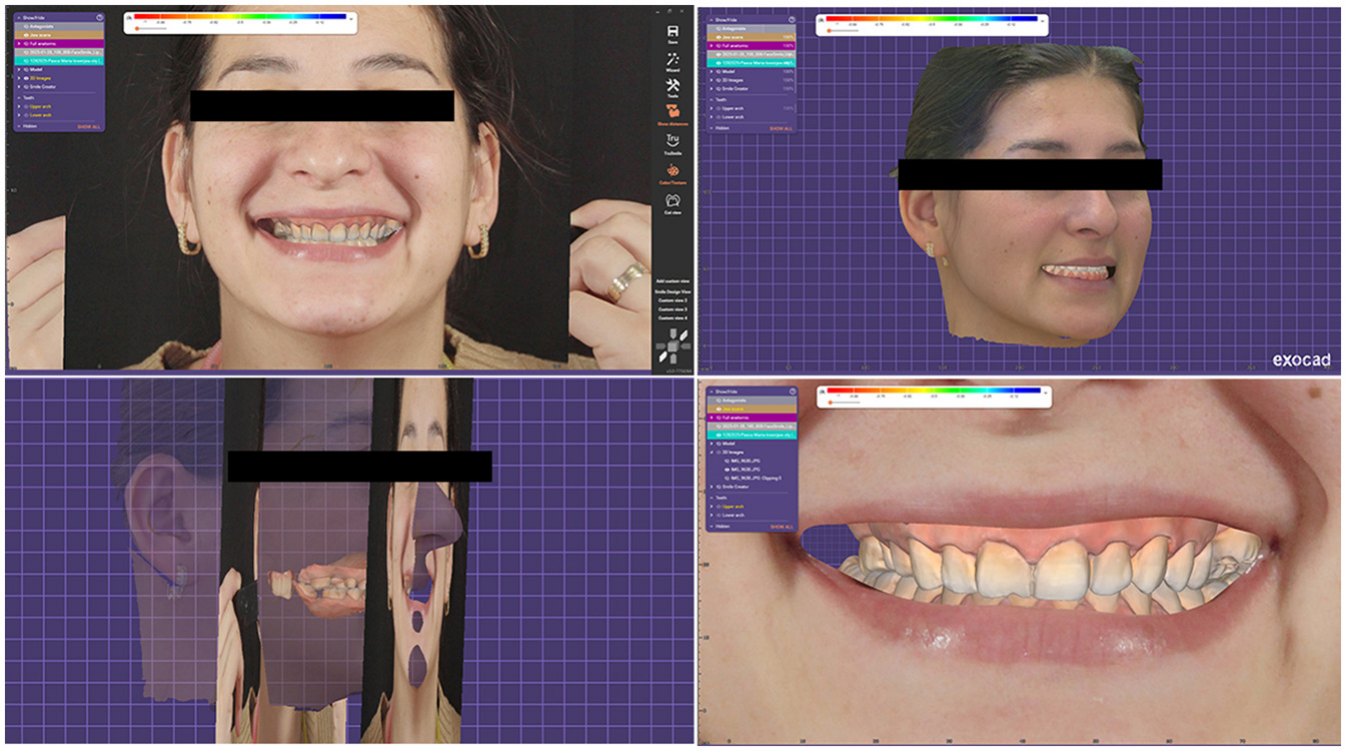
Virtual patient inside DentalCAD software.

**Figure 6:**
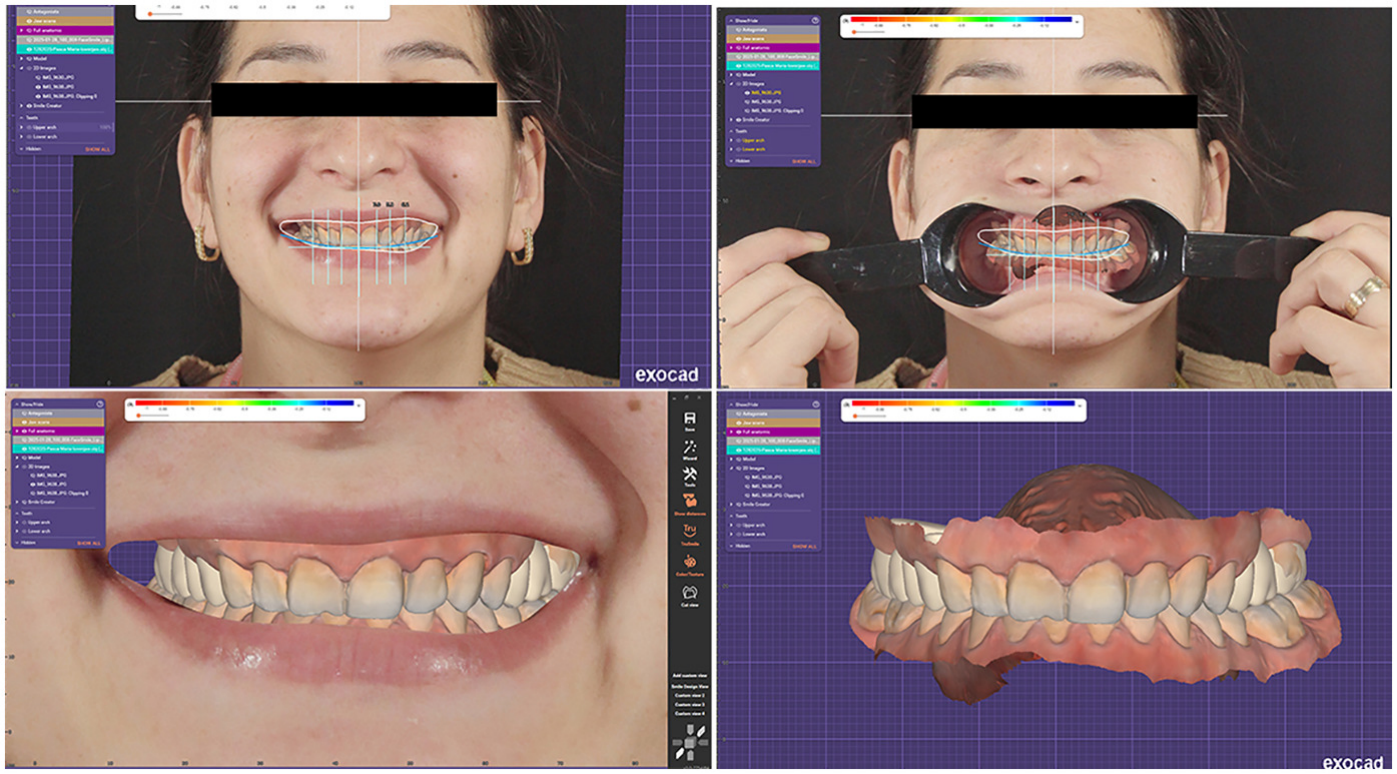
Digital smile design (DSD) inside the smile creator module.

All collected digital data were imported into DentalCAD software (exocad GmbH, Darmstadt, Germany) for planning and design (Figure 5). A Digital Smile Design (DSD) was created using the Smile Creator module, utilizing facial reference points to guide the aesthetic planning of the final restorations (Figure 6).

Following a comprehensive assessment and discussion of treatment alternatives, a fully digital workflow was selected, including interim restorations and the fabrication of milled full-contour monolithic zirconia fixed partial dentures (FPDs) (IPS e.max ZirCAD Prime, Ivoclar).

### 2.4 Digital wax-up, 3D printing, and fabrication of provisional restorations

**Figure 7:**
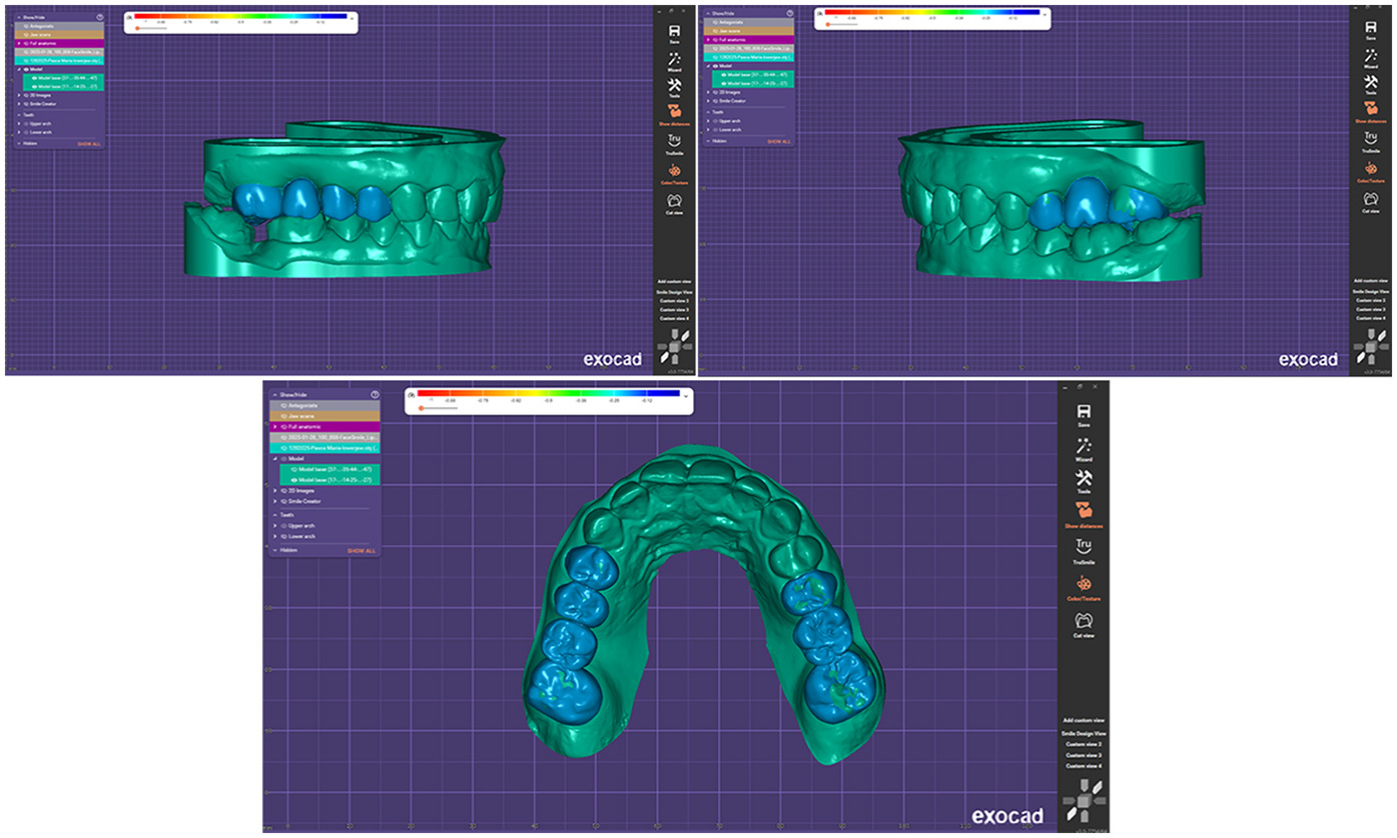
Virtual wax-up model.

**Figure 8:**
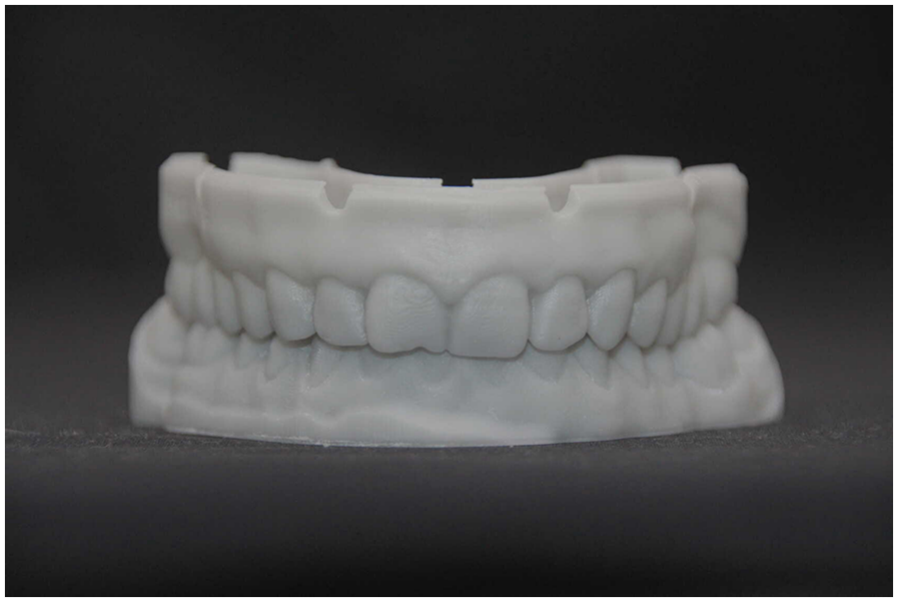
3D printed wax-up model.

Based on the diagnostic data and the Digital Smile Design, a virtual wax-up was created in DentalCAD (exocad GmbH, Darmstadt, Germany) (Figure 7). The design was then transferred to a 3D printer (Anycubic Photon M5s, Anycubic, Shenzhen, China) and printed using high-speed gray model resin (Figure 8), that was previously calibrated according to the manufacturer's specifications.

**Figure 9:**
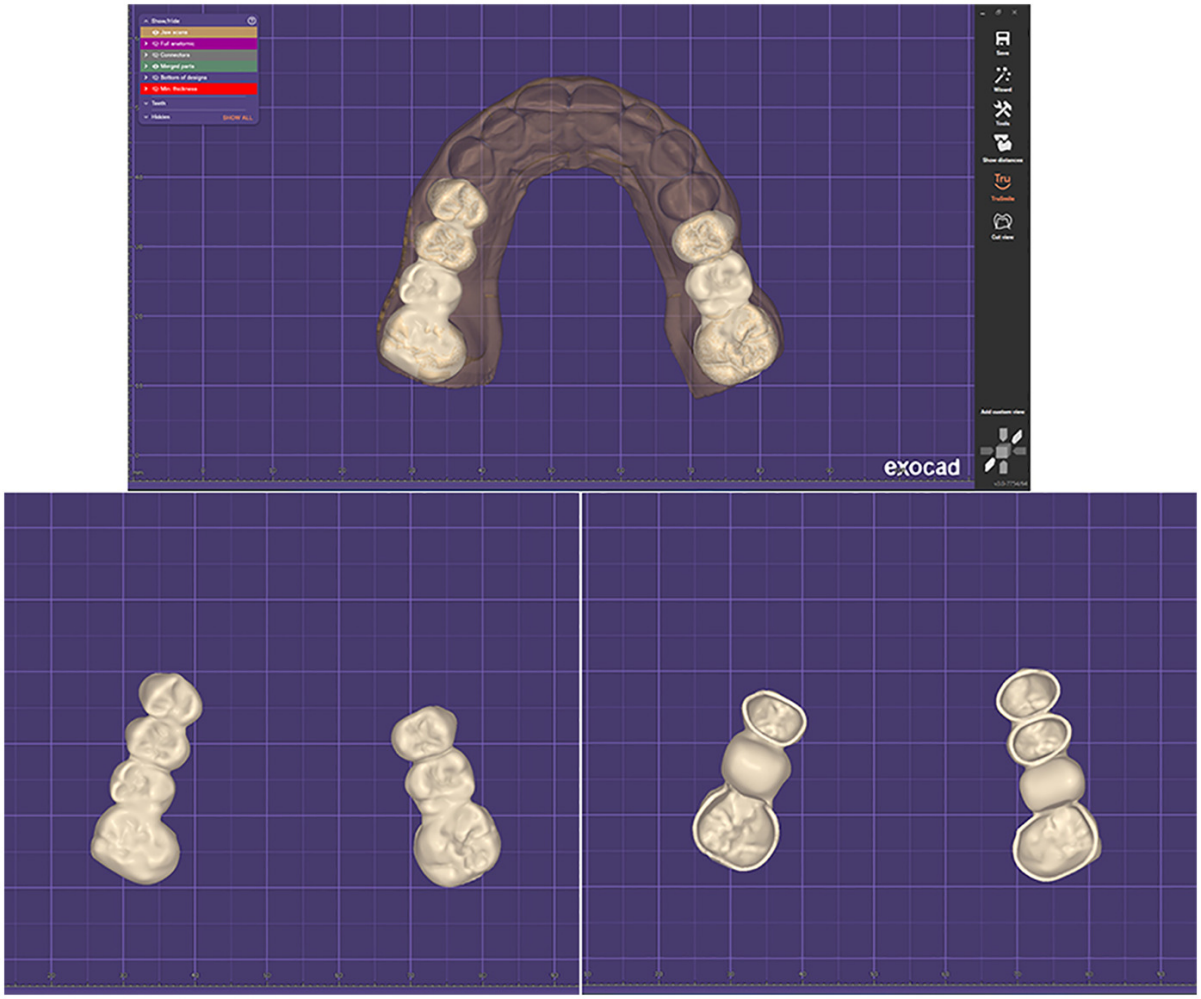
Digital design of the eggshell-type provisional restorations inside DentalCAD.

**Figure 10:**
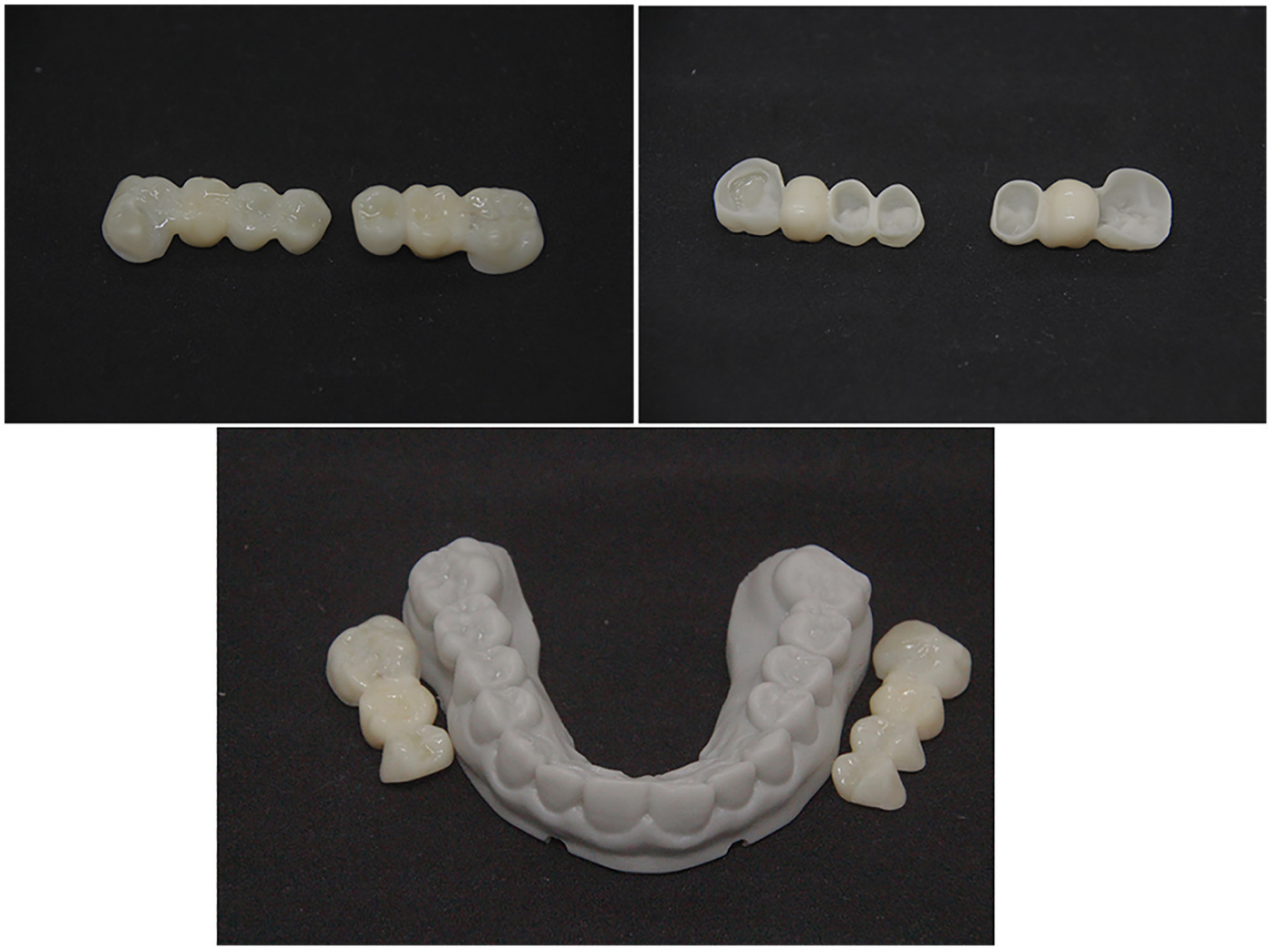
Printed eggshell-type provisional restorations.

Two eggshell-type provisional restorations were digitally designed from the wax-up to serve as indirect temporaries, to be rebased intraorally following tooth preparation. The first provisional restoration spanned teeth 1.7, 1.5, and 1.4, with tooth 1.6 as the pontic; the second included teeth 2.7 and 2.5, with tooth 2.6 as the pontic (Figure 9). These provisional shells were printed using the Prusa SL1 3D printer (Prusa Research, Prague, Czech Republic) with SprintRay Crown resin (SprintRay Inc., Los Angeles, CA, USA) (Figure 10). The printer settings were adjusted to comply with the resin's manufacturer-recommended exposure parameters.

**Figure 11:**
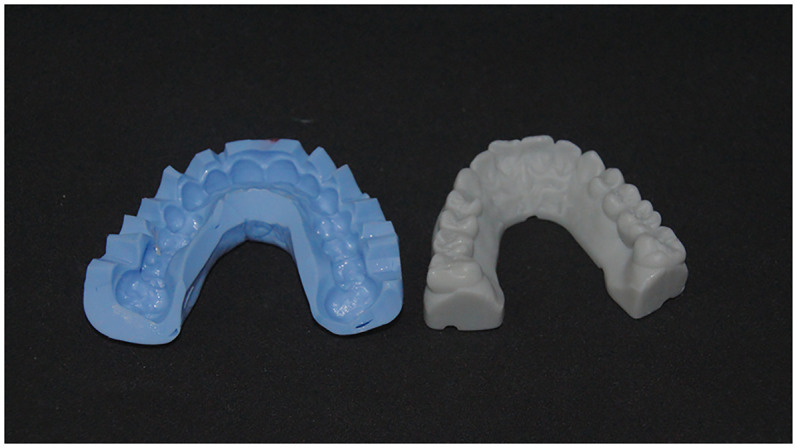
Silicon index based on the wax-up simulation.

**Figure 12:**
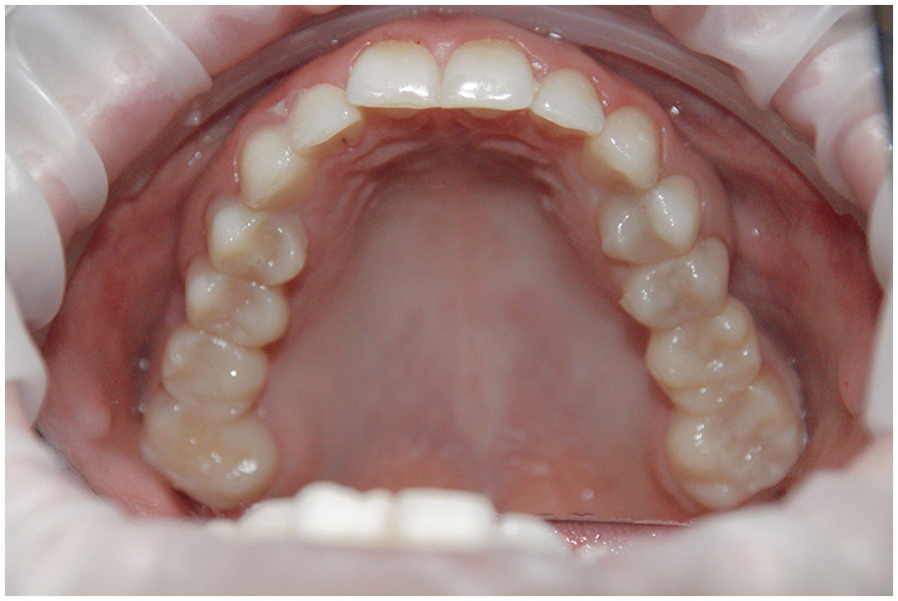
Mock-up simulation inside the patient's mouth.

To transfer the wax-up into the patient's mouth, a silicone index (Figure 11) was fabricated using addition silicone (Variotime, Kulzer GmbH, Hanau, Germany). The silicone index was filled with a bis-acrylic crown and bridge material (LuxaCrown, DMG, Hamburg, Germany) to generate the mock-up inside the patient's mouth. This simulation served both as a functional and aesthetic preview (Figure 12) and as a guide for performing tooth preparation in a minimally invasive, prosthetically driven manner.

### 2.5 Tooth preparation, gingival retraction, and digital impressions

**Figure 13:**
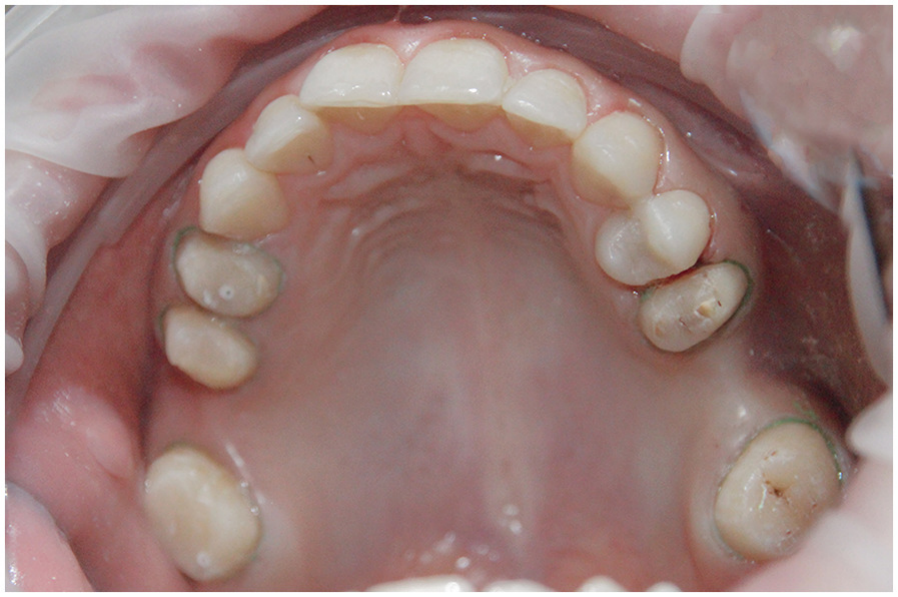
Occlusal view of the dental abutments before the digital impression.

Under local anaesthesia, the abutment teeth were prepared for full-coverage zirconia restorations using a minimally invasive approach, guided by the provisional wax-up form. Gingival displacement was performed using a single-cord retraction technique. Given the patient's thin gingival biotype, a 000-size retraction cord (Ultrapak, Ultradent Products Inc., South Jordan, UT, USA) was selected and gently placed circumferentially below the preparation margins to ensure optimal subgingival access (Figure 13).

**Figure 14:**
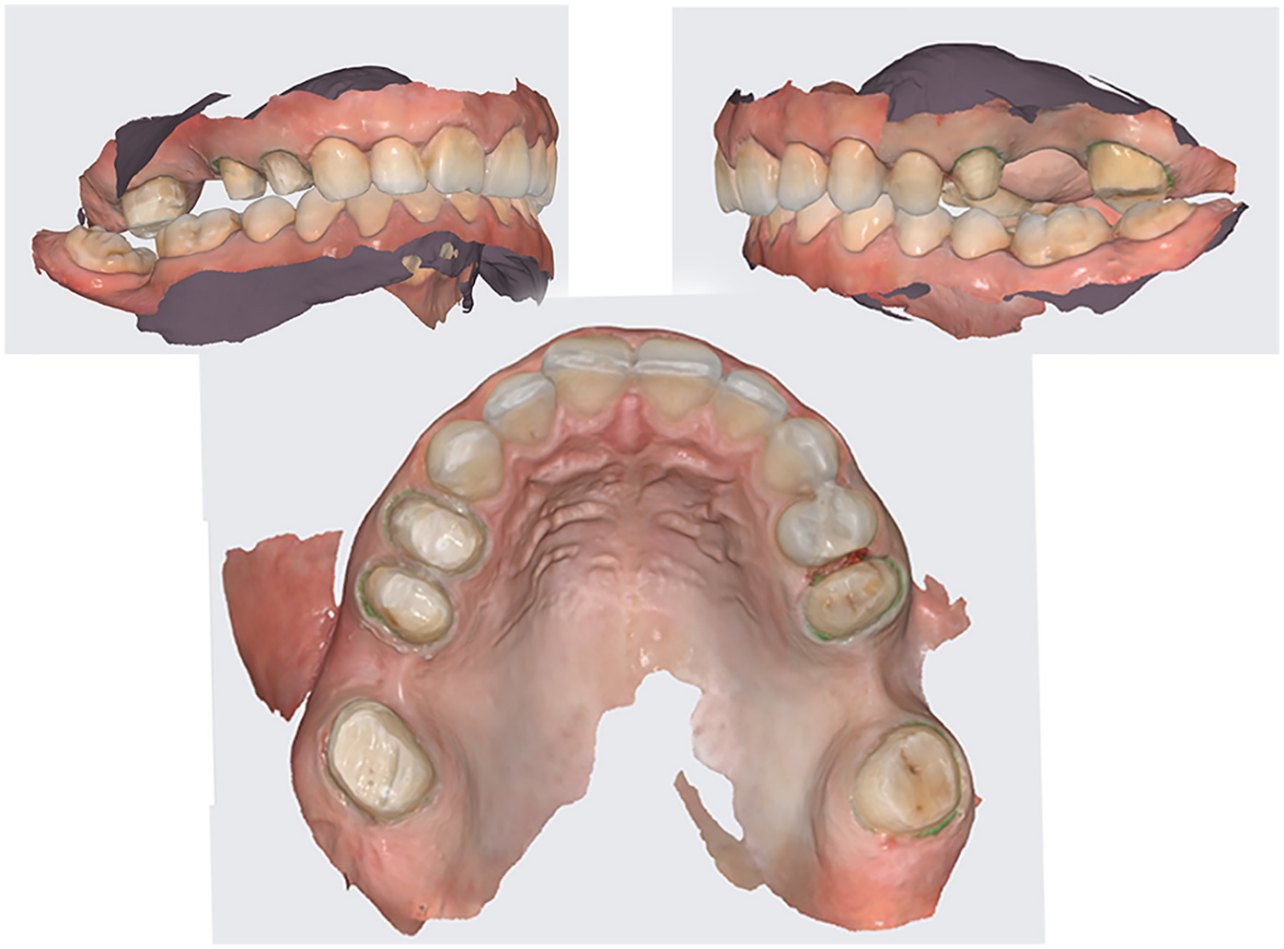
Final digital impressions of the prepared teeth.

Following gingival retraction, digital impressions of the prepared teeth were acquired using the same Medit i700 intraoral scanner (Medit Corp., Seoul, South Korea), previously calibrated per manufacturer instructions. Full-arch scans of both the maxillary and mandibular arches were completed to capture the occlusion and spatial relationships accurately (Figure 14).

**Figure 15:**
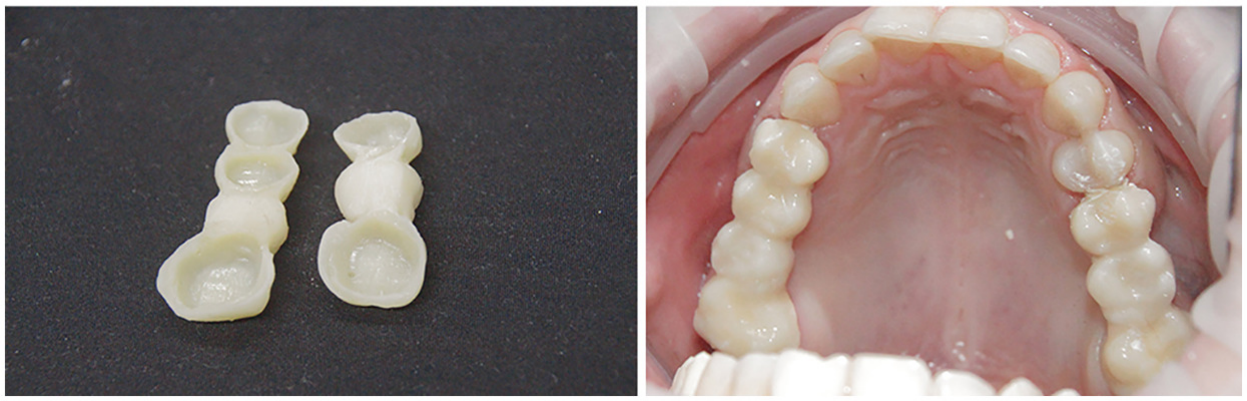
Relined eggshell provisional restorations and cementation.

The printed eggshell provisional restorations were then relined chairside using the same bis-acrylic crown and bridge material (LuxaCrown, DMG, Hamburg, Germany) and provisionally cemented using a eugenol-free temporary cement (TempBond NE, Kerr Corporation, Orange, CA, USA) (Figure 15). This ensured temporary function and protection of the abutments while the final restorations were being fabricated.

### 2.6 Laboratory workflow and fabrication of final restorations

The digital impressions, along with all preoperative data—including the Digital Smile Design, facial scan, wax-up, and photographic records—were transmitted to the dental laboratory. This comprehensive digital dataset allowed the technician to replicate the clinical situation virtually and design the final restorations with precision.

**Figure 16:**
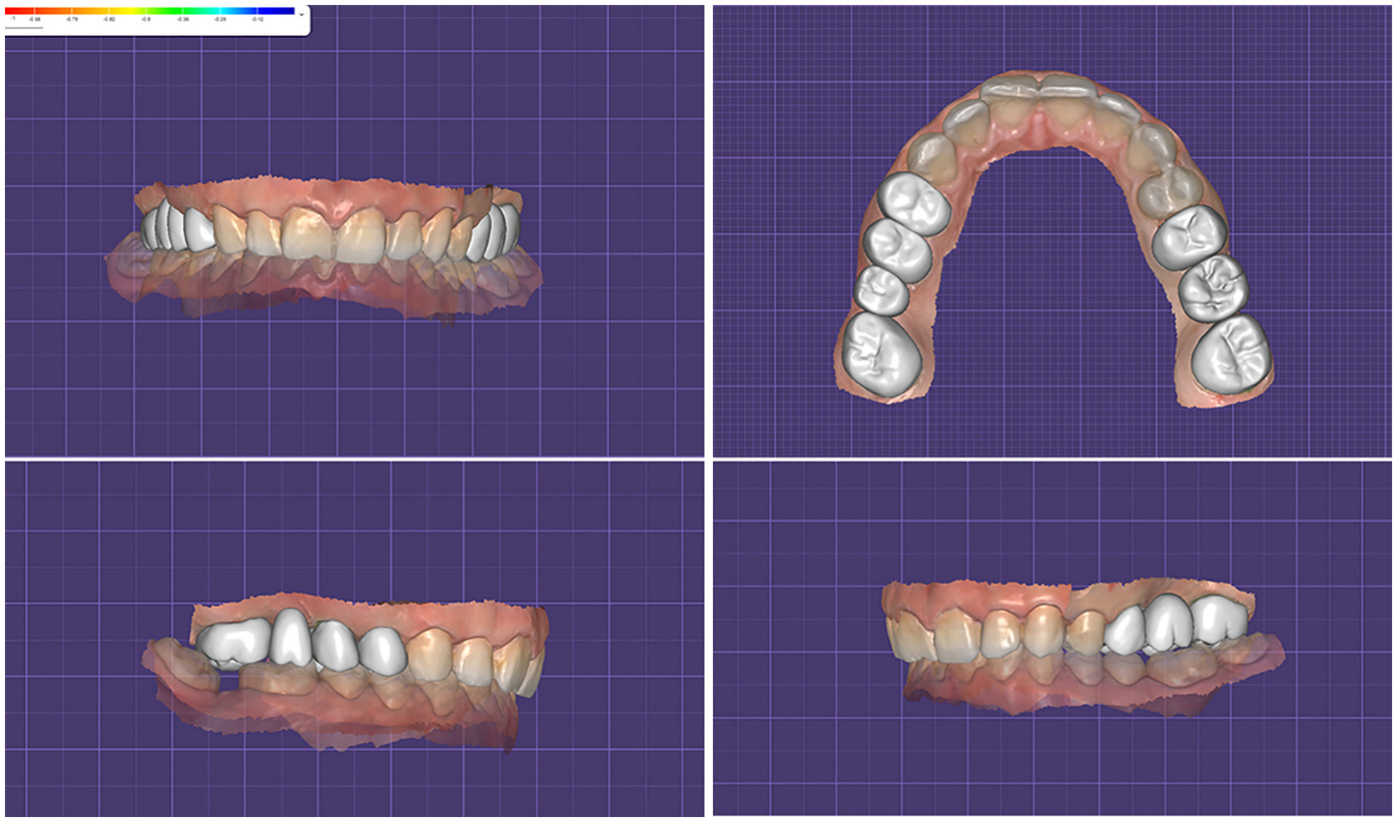
Final restorations design of the two full-contour monolithic zirconia fixed partial dentures inside DentalCAD.

Using DentalCAD (exocad GmbH, Darmstadt, Germany), two full-contour monolithic zirconia fixed partial dentures (FPDs) were digitally designed based on the previously established esthetic and functional parameters (Figure 16). The restorations were fabricated from IPS e.max ZirCAD Prime (Ivoclar Vivadent, Schaan, Liechtenstein), a high-strength, multi-layered zirconia known for its excellent mechanical properties and lifelike esthetics.

**Figure 17:**
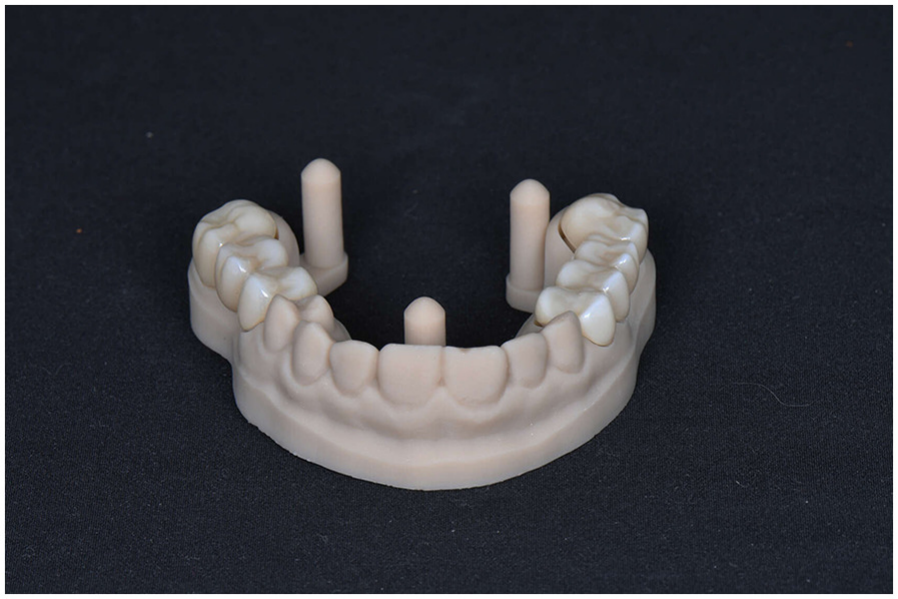
Final fixed partial dentures on the printed working model.

A 3D-printed working model was produced in the laboratory to verify fit and support the finalization of the FPDs (Figure 17). The restorations were stained on the buccal surface and polished all-around to enhance characterization but deliberately not glazed, to preserve the original occlusal morphology and contact integrity established during the digital planning phase.

**Figure 18:**
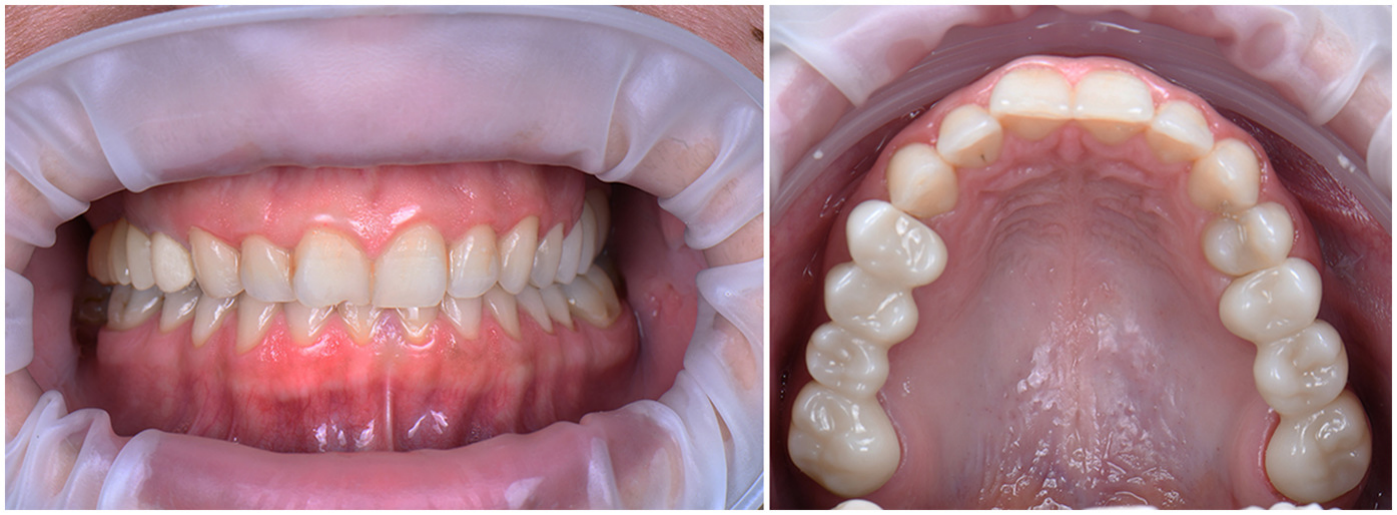
Final fixed partial dentures after cementation.

**Figure 19:**
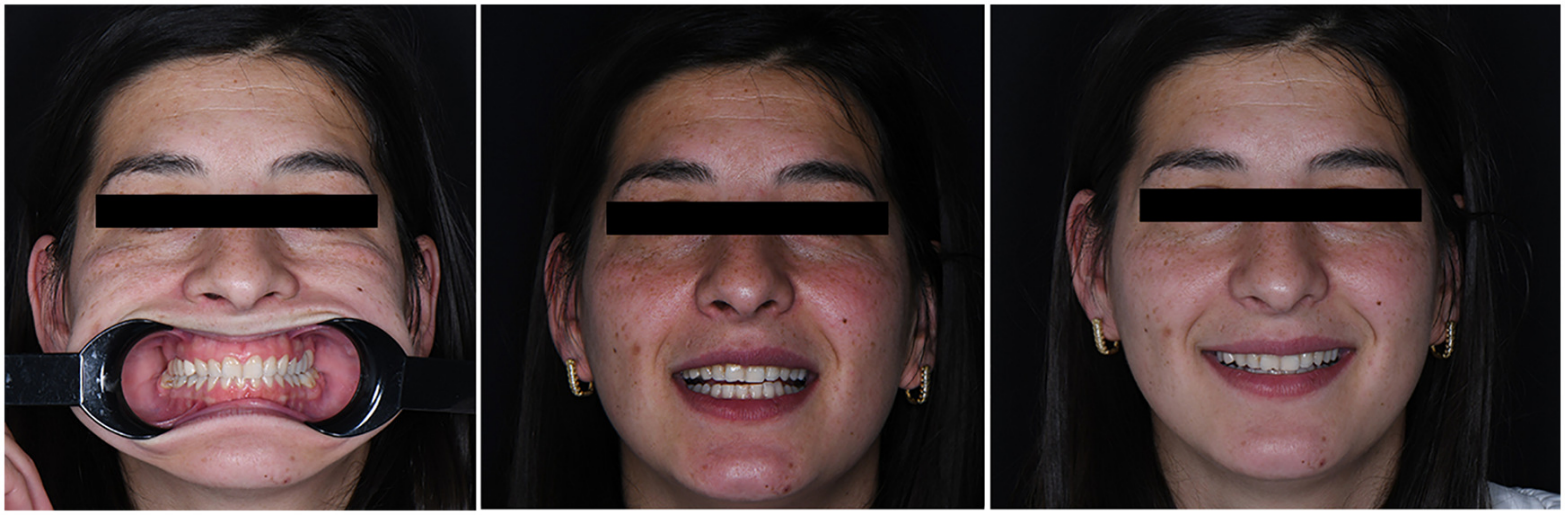
Final esthetic outcome.

The completed restorations underwent clinical try-in and were evaluated for marginal fit, esthetics, and occlusal relationships. Both the patient and the clinical team approved the final result, and the FPDs were definitively cemented using a resin-modified glass ionomer cement (GC FujiCEM 2, GC Corporation, Tokyo, Japan). The cemented restorations are shown in Figure 18, and the final esthetic outcome is presented in Figure 19.

### 2.7 Post-cementation occlusal contact analysis and comparative evaluation

After definitive cementation of the final restorations, a full-arch intraoral scan was performed using the Medit i700 (Medit Corp., Seoul, South Korea) to capture the post-treatment situation. The occlusal contact points were assessed using both digital and conventional methods to evaluate the accuracy of the digitally designed occlusion in translating to the intraoral environment. All occlusal evaluations and adjustments were performed by the same experienced clinician to ensure consistency and reduce operator variability.

**Figure 20:**
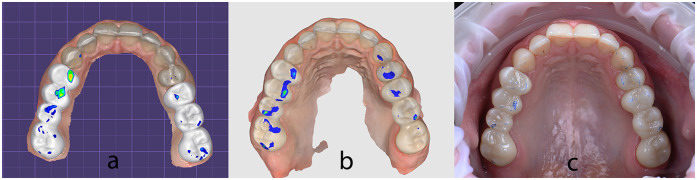
Occlusal contact points captured: **(a)** inside DentalCAD on the final restorations aligned with the opposing arch; **(b)** inside medit occlusion analyzer on the new post-cementation intraoral scan; **(c)** clinically inside the patient's mouth using 40 µm articulating paper.

Initially, in the virtual environment, the expected occlusal contacts were analysed within the CAD design software (DentalCAD, exocad GmbH) on the final restorations aligned with the opposing arch (Figure 20a). Subsequently, the Medit Occlusion Analyzer tool was used to assess occlusal contacts based on the new post-cementation intraoral scan. This allowed for a digital visualization and documentation of contact points within the patient's mouth (Figure 20b).

For physical validation, 40 µm articulating paper (BK80, Bausch GmbH, Cologne, Germany) was used intraorally with the patient in maximum intercuspation to mark static contact points (Figure 20c). These markings were photographed and compared to the digitally captured occlusal maps.

**Figure 21:**
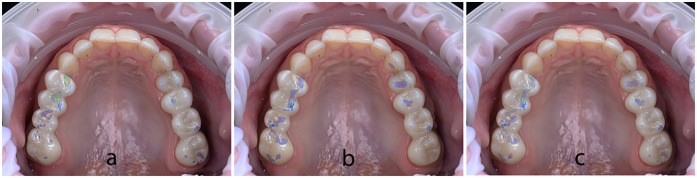
**(a)** DentalCAD contacts superimposed over intraoral articulating paper photo; **(b)** occluzion analyzer contacts superimposed over intraoral articulating paper photo; **(c)** all three visualizations superimposed.

All three visualizations—from DentalCAD, Medit Occlusion Analyzer, and intraoral articulating paper—were compared side by side to assess the transfer accuracy of the digitally designed contacts into the final intraoral restorations (Figure 21). A qualitative, visual comparison of the contact positions revealed a level of consistency between the CAD-planned contacts and those observed clinically. The position of most of the contacts remained stable across all evaluation methods. However, some variations were noted in the intensity of the contacts, particularly in the digital visualizations. While the articulating paper confirmed positional accuracy, only the digital tools (Exocad and Medit Occlusion Analyzer) provided insights into the force distribution and relative pressure intensity of the contacts.

### 2.8 Dynamic occlusion evaluation and final functional assessment

To gain a comprehensive understanding of both static and dynamic occlusion, a final occlusal analysis was performed using the OccluSense system (Bausch GmbH, Cologne, Germany). This device integrates pressure-sensitive electronic sensors with traditional ink-based occlusal marking, providing both visual and quantitative data on contact distribution and occlusal force.

**Figure 22:**
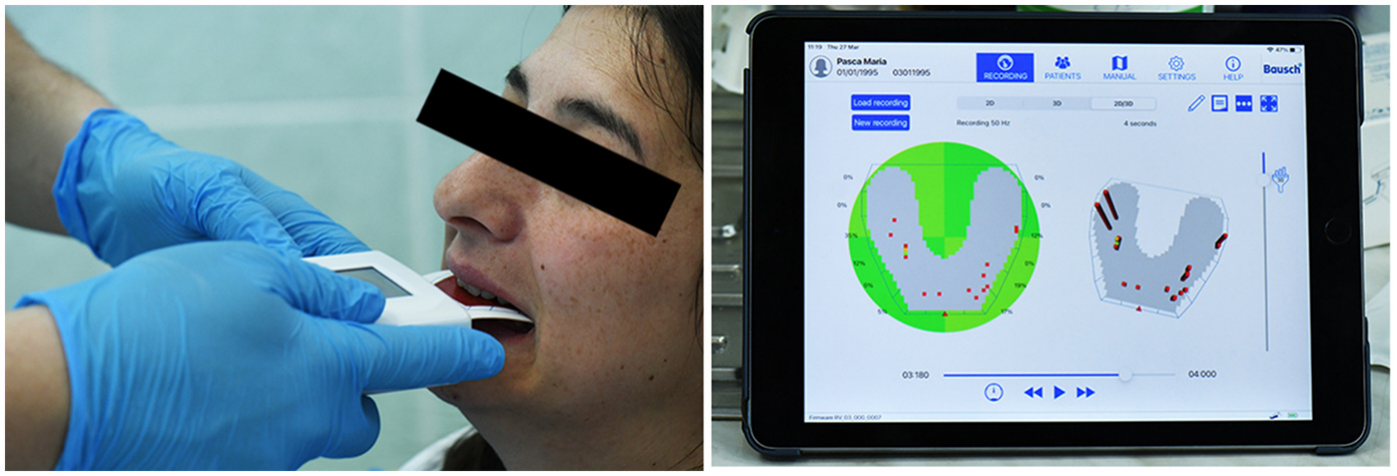
Occlusense occlusal analysis.

The OccluSense sensor was positioned intraorally, and the patient was guided through various mandibular movements, including centric occlusion, protrusion, and lateral excursions. Data were wirelessly transmitted to the dedicated OccluSense iPad application, where occlusal contacts were visualized in full color (Figure 22), and pressure distribution was presented as a percentage for each tooth contact.

**Figure 23:**
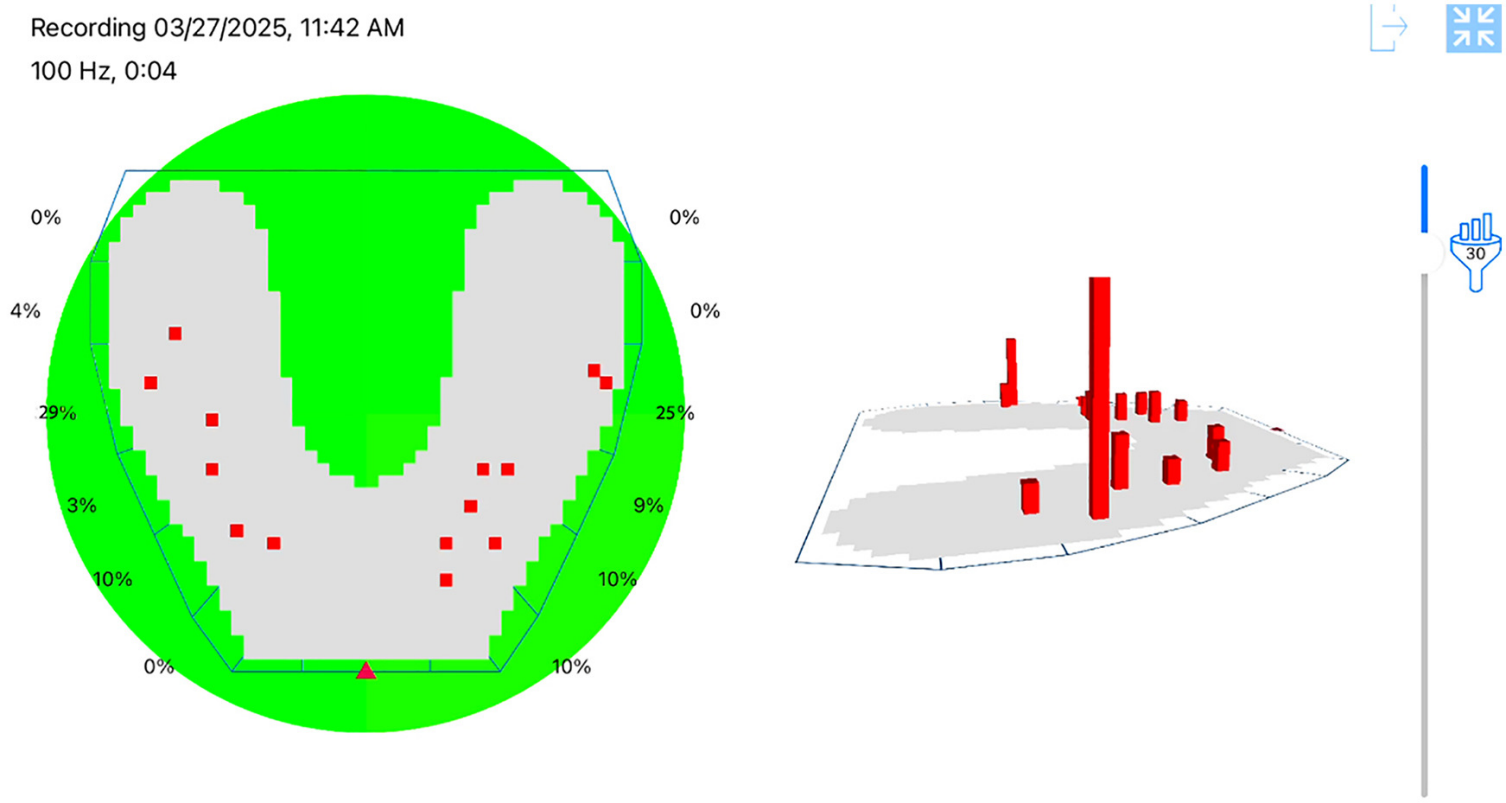
Stable and balanced occlusion in maximum intercuspation presented inside OccluSense occlusal analysis.

**Figure 24:**
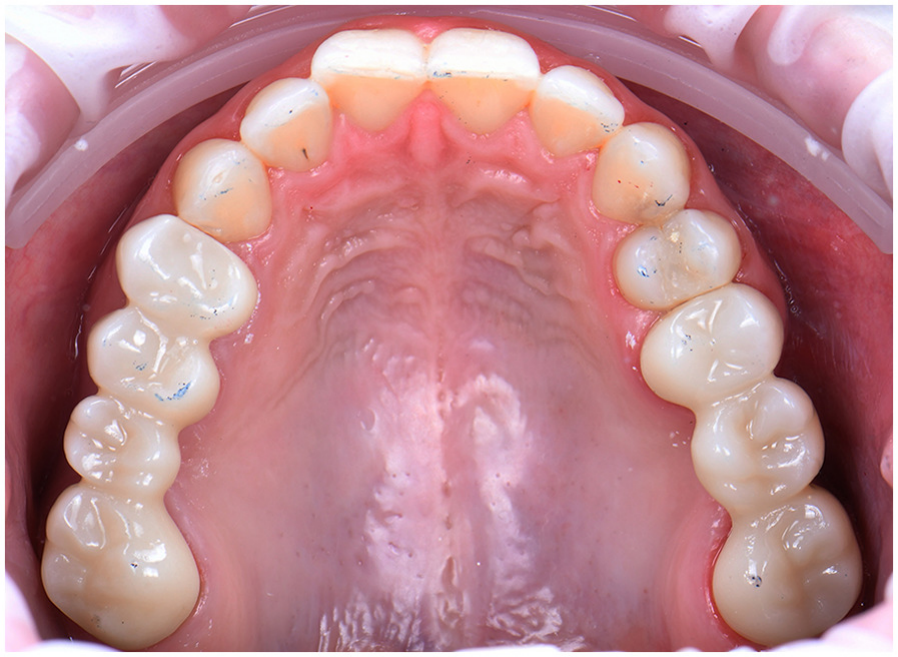
Final occlusal contacts in maximum intercuspation after occlusal adjustments, marked with 40 µm articulating paper, intraoral photo.

The analysis confirmed that the patient exhibited stable intercuspal contacts and occlusal harmony in maximum intercuspation (Figure 23). However, dynamic evaluation revealed premature occlusal contacts during right laterotrusion. These interferences were precisely identified using the force distribution data and were subsequently corrected through selective enamel grinding, following the digital guidance provided by the system (Figure 24).

This data-driven approach allowed for refined occlusal adjustments, improving functional balance and minimizing the risk of long-term complications such as muscle strain or restoration overload. The patient reported immediate improvement in comfort during mastication following the adjustment phase.

**Figure 25:**
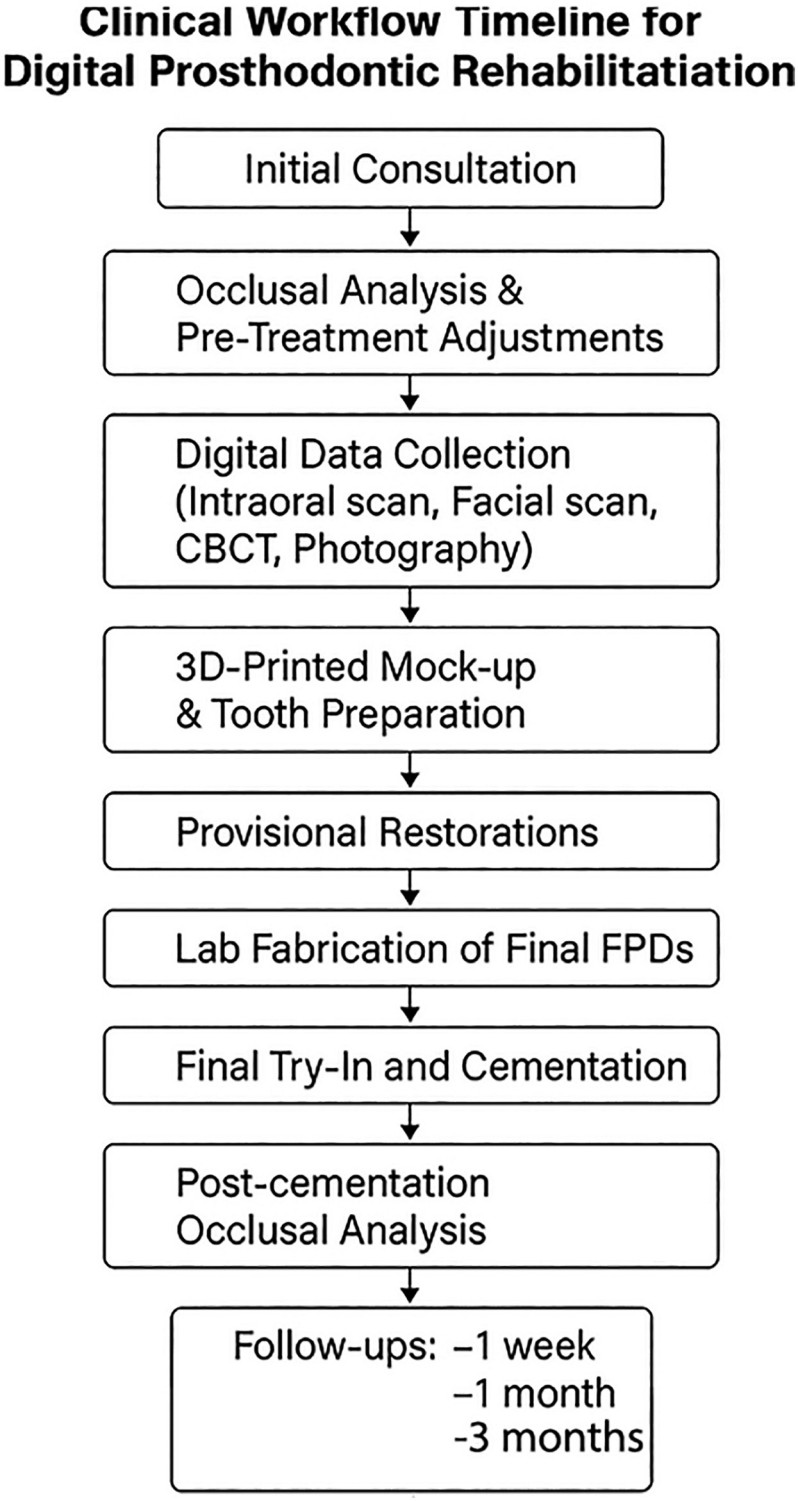
Clinical workflow timeline for digital prosthodontic rehabilitation.

To further clarify the treatment chronology, a visual representation of the clinical workflow has been included (Figure 25). This timeline summarizes the sequential integration of digital tools and clinical procedures, from initial assessment to final follow-up, illustrating the structured and reproducible nature of the fully digital prosthodontic rehabilitation protocol.

### 2.9 Follow-up and clinical outcome

The patient was scheduled for follow-up visits at 1 week, 1 month, and 3 months post-cementation. At each recall, a comprehensive clinical evaluation was performed, focusing on the integrity of the restorations, occlusal stability, soft tissue health, esthetic integration, and patient-reported outcomes.

At the 1-week follow-up, the patient reported no discomfort during mastication and expressed satisfaction with the overall appearance and feel of the restorations. Visual and tactile inspection confirmed proper marginal adaptation, with no evidence of cement washout or irritation to the surrounding gingiva. Occlusal contacts remained stable, and no further adjustments were required.

By the 1-month follow-up, the restorations had integrated harmoniously into the patient's dentition. Periodontal tissues around the abutments appeared healthy, with no signs of inflammation or recession. The occlusal scheme remained balanced and free of premature contacts, as verified with articulating paper and patient feedback during chewing simulation. Esthetically, the zirconia restorations exhibited excellent color matching and surface luster. The patient reported improved comfort and function compared to the provisional phase.

At the 3-month recall, the prostheses continued to perform successfully, with no reported functional limitations, chipping, or debonding. The gingival margins remained stable with good tissue adaptation, and oral hygiene was well maintained. The occlusion continued to demonstrate stability in both centric and eccentric movements. The patient expressed high satisfaction with the treatment outcome, particularly noting the natural appearance of the restorations and the absence of any postoperative complications. No adverse events or prosthetic complications were observed during the entire follow-up period.

## 3 Discussion

The advancement of digital technologies in dentistry has enabled the implementation of predictable and reproducible workflows for prosthetic rehabilitation, especially in complex cases involving multiple-unit restorations (9, 10). In the present case, the comprehensive use of intraoral scanning, facial scanning, cone-beam computed tomography (CBCT), and digital occlusal analysis facilitated a minimally invasive, patient-centered approach with enhanced precision throughout all stages of treatment.

A key feature of this case was the successful integration and superimposition of various digital datasets to create a fully digitized patient model. This digital fusion allowed for precise planning of tooth preparations, provisional restoration design, and definitive prosthetic fabrication—all while preserving existing healthy structures and minimizing chairside time.

The fabrication of provisional restorations via 3D printing played an important role in both aesthetic and functional previewing. These interim restorations were designed from a digital wax-up and served not only as a clinical mock-up but also as a guide for minimally invasive tooth preparation. This step also enabled real-time patient feedback, enhancing patient involvement and satisfaction in the treatment process (7).

Traditionally, occlusal assessment relies on articulating paper, a method influenced by material variability and clinician subjectivity. While articulating paper remains widely used, its limitations are well documented, especially in distinguishing contact intensity and dynamic interferences (16). In this case, the combination of traditional and digital occlusal analysis techniques proved highly effective. The OccluSense® system allowed for the objective quantification of occlusal contacts and forces, providing real-time, color-coded data on pressure distribution during both static and dynamic movements (17, 18). Recent clinical studies have also demonstrated the utility of digital occlusal analysis tools, including OccluSense, in evaluating contact precision and validating CAD-based prosthetic planning in implant-supported restorations (19, 20). This data was essential in diagnosing and eliminating premature contacts—particularly on the mandibular third molars—which could have otherwise compromised the long-term stability of the prosthetic restorations and patient comfort.

All three visualizations—DentalCAD software, Medit Occlusion Analyzer, and intraoral articulating paper—were qualitatively compared to assess the accuracy of occlusal contact transfer. This multi-modal approach enabled a visual triangulation of static contact positions, although it did not involve force-calibrated measurements in this phase of the analysis. However, a digital pressure-based occlusal analysis was conducted at the end of treatment using the OccluSense system, which combines electronic force sensors with traditional ink-based markers. While not directly integrated into the CAD-based occlusal planning validation, OccluSense was instrumental in identifying premature contacts during dynamic mandibular movements and guided the final occlusal refinements. Future studies could enhance this workflow by incorporating OccluSense or similar systems such as T-Scan during both the planning and validation phases, allowing for a comprehensive, quantitative analysis of occlusal force distribution and timing. This level of precision is crucial in preventing temporomandibular joint disorders and maintaining long-term occlusal stability (21).

One notable challenge in this digital workflow was the absence of integrated mandibular motion registration. While most of the occlusal contact points planned in the CAD environment translated accurately intraorally, minor discrepancies—particularly during right laterotrusion—were observed. These variations were attributed to the lack of dynamic occlusion data in the virtual planning phase. Incorporating digital jaw tracking systems (such as Zebris or Modjaw) or advanced virtual articulators that simulate mandibular movements could significantly enhance predictive accuracy. These technologies allow integration of real-time kinematic data, enabling more realistic occlusal mapping and potentially reducing the need for post-cementation adjustments.

The digital impressions acquired using the Medit i700 intraoral scanner demonstrated excellent accuracy, as verified through reference scan alignment and surface deviation analysis (22, 23). The resulting restorations required no occlusal surface modifications, supporting the scanner's reliability in capturing fine details. Furthermore, the decision to avoid glaze application on the zirconia restorations maintained the integrity of occlusal contact morphology, ensuring that contacts were preserved as designed.

This case also underlines the clinical utility of an interdisciplinary and technology-driven approach, particularly in bilateral posterior rehabilitations where occlusal balance is critical (24, 25). The use of a fully digital workflow enabled high precision, reduced chairside time, and improved communication between clinicians and dental technician. However, such workflows demand a high level of technical proficiency, and there remains a learning curve for practitioners transitioning from conventional to digital systems.

Despite the overall success of the digital workflow, a minor aesthetic limitation was observed in the final outcome. Specifically, the delivered zirconia restorations exhibited slight buccolingual over-contouring, as visible in the postoperative extraoral images. While this did not affect functional performance or occlusal harmony, such contouring may compromise the perceived naturalness of the restorations in certain clinical views. In the second quadrant, it is important to note that the abutment teeth (2.5 and 2.7) were vital and thus prepared in a minimally invasive manner to preserve pulp vitality. As a result, the final prosthetic design may have necessitated slightly more material volume to achieve proper coverage and retention, contributing to the observed bulkiness. The aesthetic outcome was nonetheless reviewed and approved by the patient prior to definitive cementation and corresponded closely with the Digital Smile Design (DSD) simulation. Future digital workflows could benefit from enhanced control over buccal emergence profiles, potentially through more refined 3D design protocols or AI-driven morphology validation. Addressing such subtle contour discrepancies may further improve aesthetic predictability, particularly in cases involving high smile lines or anterior transitions.

Further research involving larger population sample, longer follow-up periods, and integration of dynamic jaw tracking is warranted to validate the reproducibility and long-term success of comprehensive digital protocols (14). Integration of jaw motion tracking systems, such as digital axiography or virtual articulators powered by real-time kinematic data, into the restorative CAD workflow, would allow for improved prediction of dynamic occlusal behaviour and may significantly reduce the need for post-cementation occlusal adjustments. Additionally, the use of artificial intelligence for occlusal contact analysis and force simulation may open new pathways for automation and standardization of digital prosthodontic treatment (17).

## 4 Conclusion

This clinical case report illustrates the successful implementation of a fully digital workflow for the prosthetic rehabilitation of a patient requiring fixed dental prostheses in the posterior maxilla. The treatment plan, selected in agreement with the patient who declined implant therapy, focused on delivering functional and aesthetic restorations using state-of-the-art digital technologies.

Each phase—from diagnosis, planning, and provisional restorations to final fabrication and post-cementation evaluation—was executed with the aid of integrated digital tools including intraoral scanning, facial scanning, CAD software, 3D printing, and digital occlusion analysis systems. While a high degree of consistency was observed between the virtually planned occlusal contacts and those identified intraorally, some discrepancies were noted. Certain contact points were either missing or slightly displaced but remained within the same tooth or adjacent occlusal area. Additionally, a few dynamic interferences were present post-cementation, which could not be anticipated, as the digital workflow did not incorporate jaw motion tracking or dynamic occlusal simulation.

The use of advanced occlusal analysis tools, such as the OccluSense system, played a critical role in identifying and correcting these interferences, ultimately achieving a functional occlusal scheme in both static and dynamic conditions. These refinements contributed to improved patient comfort and the long-term success of the restorations.

This case highlights the potential of digital dentistry to provide predictable, efficient, and less time chairside adjustments for the fixed dental prostheses. Nonetheless, it also underlines the current limitations of static-only digital planning and the potential value of incorporating dynamic mandibular motion into future digital workflows. Further clinical studies are warranted to assess the consistency and clinical impact of fully digital protocols across broader patient populations.

## Data Availability

The original contributions presented in the study are included in the article/Supplementary Material, further inquiries can be directed to the corresponding author.
